# Coconut husk biochar amendment enhances nutrient retention by suppressing nitrification in agricultural soil following anaerobic digestate application^☆^

**DOI:** 10.1016/j.envpol.2020.115684

**Published:** 2021-01-01

**Authors:** Jidapa Plaimart, Kishor Acharya, Wojciech Mrozik, Russell J. Davenport, Soydoa Vinitnantharat, David Werner

**Affiliations:** a School of Engineering, Newcastle University, Newcastle Upon Tyne, NE1 7RU, United Kingdom; b Environmental Technology Program, School of Energy, Environment and Materials, King Mongkut’s University of Technology Thonburi, Bangkok, 10140, Thailand

**Keywords:** Agricultural waste, Anaerobic digestate, Biochar, Nitrification, Nutrient pollution

## Abstract

Anaerobic digestate and biochar are by-products of the biogasification and pyrolysis of agricultural wastes. This study tested the hypothesis that combined application of anaerobic pig/cattle manure digestate and coconut husk (CH) biochar can improve soil nutrient conditions, whilst minimizing atmospheric and groundwater pollution risks. Microcosms simulated digestate application to agricultural soil with and without CH biochar. Ammonia volatilization and nutrient leaching were quantified after simulated heavy rainfalls. Archaeal and bacterial community and abundance changes in soils were quantified via next generation sequencing and qPCR of 16S rRNA genes. Nitrifying bacteria were additionally quantified by qPCR of functional genes. It was found that CH biochar retarded nitrate leaching via slower nitrification in digestate-amended soil. CH biochar reduced both nitrifying archaea and bacteria abundance in soil by 71–83 percent in the top 4 cm soil layer and 66–80 percent in the deeper soil layer one month after the digestate application. Methanotroph abundances were similarly reduced in the CH biochar amended soils. These findings demonstrate combined benefits of anaerobic digestate and CH biochar application which are relevant for the development of a more circular rural economy with waste minimization, renewable energy production, nutrient recycling and reduced water pollution from agricultural land.

## Highlights

• Combined benefits of digestate and biochar application to soil were studied.
• Microbial community response was monitored via next generation sequencing and qPCR.
• Coconut husk biochar amendment slightly enhanced nutrient adsorption.
• Biochar reduced nitrifier abundance and nitrification in digestate-amended soil.
• Nutrients from biowastes were recycled with reduced groundwater pollution risks.

## Graphical abstract

**Figure:**
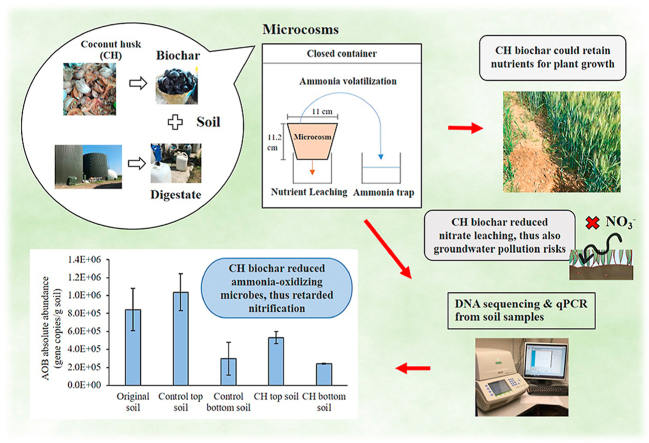

## Teaser

**Capsule:** Chemical and molecular microbiology measurements elucidated biochar and anaerobic digestate effects on nutrient retention and nitrification in soil, and groundwater pollution risks.

## 1 Introduction

Due to the rapidly increasing world population and urbanization, global demand for livestock products is expected to double by 2050, particularly in Asia, Africa and Latin-America (Scholten et al., 2013). Consequently, there will be a significantly higher number of livestock farms with waste generation as animal manure and slurry. These readily biodegradable agricultural wastes can be transformed into biogas through anaerobic digestion (Holm-Nielsen et al., 2009). Anaerobic digestion also creates a nutrient-rich liquid by-product known as anaerobic digestate (Holm-Nielsen et al., 2009). The digestate can be used as a bio-fertilizer and save farmers the cost of artificial fertilizers (Lukehurst et al., 2010). According to previous research, digestate provides higher potential benefits for nitrogen (N) availability and crop yields compared to untreated animal manures (Möller and Müller, 2012). However, there have been concerns over ammonia volatilization and nutrient leaching from soils to groundwater after digestate application (Lukehurst et al., 2010). This is attributed to rapid ammonification of organic nitrogen followed by nitrification of ammonia into the more soluble and leachable nitrogen compound, nitrate (Wang et al., 2015). About 50–70% of nitrogen in fertilizer may be lost to nitrification related processes (Singh and Verma, 2007). Nitrification involves the oxidation of nitrogen compounds in a two-step process in which ammonia is first oxidized to nitrite by ammonia-oxidizing bacteria (AOB), e.g. *Nitrosomonas* and *Nitrosospira*, and ammonia-oxidizing archaea (AOA). Subsequently, nitrite is converted to nitrate by nitrite-oxidizing bacteria (NOB), e.g. *Nitrobacter* and *Nitrospira* (Singh and Verma, 2007; Wang et al., 2015; Han et al., 2018). Some *Nitrospira* species are also capable of oxidizing ammonia to nitrate on their own in both, water and soil systems (Pjevac et al., 2017).

Water pollution control and nutrient recovery via adsorption is feasible using a wide variety of biosorbents derived from waste biomass (Takaya et al., 2016). Biochar, a carbon-rich material, is one of these biosorbents. It is produced by heating biomass feedstock such as wood and agricultural waste through pyrolysis or biogasification for renewable energy generation (Cole et al., 2012). Different feedstock sources and pyrolysis process conditions contribute to different structural and physical characteristics of biochar including structural complexity, surface area, porosity, particle size distribution, density and mechanical strength (Lehmann and Joseph, 2015). Biochar can play an important role in enhancing nutrient retention in soil mostly due to its surface charge density (Kongthod et al., 2015). Biochar mostly has negatively charged surfaces which increases the adsorption capacity of cation species (Lou et al., 2016). Biochar has gained interest in the multidisciplinary areas of global warming mitigation, soil amendment, crop production enhancement and carbon sequestration (Glaser et al., 2002; Laird, 2008; Tan et al., 2015). Biochar has great potential for improving soil fertility (Ahmad et al., 2014). This can be partially attributed to effects on soil microbiology that reduce fertilizer losses via leaching (Atkinson et al., 2010; Tan et al., 2015).

In past decades, nitrogen-related problems and their remediation have preoccupied many researchers. Several strategies such as using slow-release fertilizers and the addition of synthetic nitrification inhibitors to fertilizer have been investigated to reduce the risk of nitrate leaching and improve N-use efficiency in agricultural systems (Singh and Verma, 2007; Lu et al., 2019). However, nitrification inhibitors are considered too expensive for large-scale applications and nitrification inhibitors synthesized from chemical compounds may also cause phytotoxicity problems (Zerulla et al., 2001). Several studies have shown that the nitrification process in soil could be altered by biochar amendment due to its effect on soil geomicrobiology (DeLuca et al., 2006; Song et al., 2014; Bi et al., 2017). Wang et al. (2015) found that nitrification was retarded by peanut shell biochar amendment in an acidic orchard soil. The utilization of biochar as a nitrification inhibitor could be a promising option for N-management in agriculture, which would be particularly relevant in co-application with a rich source of reduced nitrogen such as anaerobic digestate. Such a co-application would facilitate multi-use systems of waste by integrating two residues (biochar and digestate) of bioenergy generation from different types of agricultural waste for re-use in sustainable agriculture. There have been reports on the effect of biochar or digestate application alone on soil microbiology (Anderson et al., 2011; Xu et al., 2016a; Gielnik et al., 2019) and on the impact of combined biochar and digestate application in soil on aspects such as greenhouse gas reduction, carbon sequestration, plant growth and microbial respiration (Marchetti et al., 2012; Martin et al., 2015; Mukherjee et al., 2015; Udall et al., 2017; Cardelli et al., 2018). However, very little is known regarding the soil microbial community response, especially nitrification, following combined application of digestate and biochar.

The main aim of this work was therefore to investigate the effect of combined application of digestate with CH biochar on nutrient retention, nitrification and nitrifying bacteria and archaea abundance in an agricultural soil. It was hypothesized that the combined application i) enhances nutrient sorption, ii) reduces nutrient leaching and iii) ammonia volatilization, iv) and the abundance of nitrifying bacteria, and thereby v) the rate of nitrification in soil. These hypotheses were tested by conducting batch experiments on biochar and soil sorption, ammonia volatilization and leaching experiments using soil microcosms, and molecular analysis of soil microbial communities.

## 2 Materials and methods

### 2.1 Biochar production

This project was initiated as part of a UK-Thai collaborative investigation into the valorization opportunities for coconut husk (CH) biochar produced by an inexpensive oil drum kiln method that is accessible to low income farmers. The details of biochar production at Kasetsart University/KMUTT University, in Thailand, and CH biochar physicochemical properties are provided in supplementary information (SI).

### 2.2 Sampling of soil and digestate

Due to foreign soil and biohazardous waste import restrictions, anaerobic dairy/pig slurry digestate and an agricultural clay loam soil were obtained from Cockle Park farm in the UK. However, biogas technology is nowadays also well developed in the Thai swine farm industry (Wongsapai et al., 2008), and clay loam is a common soil type in Thailand (Tsubo et al., 2007). More details of the soil and digestate are provided in SI.

### 2.3 Characterization of digestate

The digestate pH and nutrient characteristics were determined using spectrophotometric methods detailed in SI. In addition, a synthetic digestate solution was prepared for the sorption experiments (Section 2.4) to facilitate mass balance and sorption coefficient calculations in a well-defined system. The synthetic digestate was prepared from NH_4_Cl, NaNO_3_, NaNO_2_, urea and Na_2_HPO_4_ salts as explained in SI, Table S1, based on typical digestate nutrient characteristics (Kizito et al., 2015; AHDB, 2017). The characteristics of the digestate and synthetic digestate solution used in this study were comparable with literature reports (Table 1).

**Table 1:** Comparison of nutrient characteristics and pH values of real digestate used in this study, digestate reported in literatures and a synthetic digestate solution used for sorption batch experiments in this study. Results (Mean ± S.D.) are reported to three significant figures.

Parameter	Unit	Real digestate used in this study	Real digestate reported values (Kizito et al., 2015; AHDB, 2017)	Synthetic digestate solution
NH_4_^+^-N	(mg N/L)	1630 ± 298	1390–1450	1410 ± 73.7
NO_3_^−^-N	(mg N/L)	135 ± 20.5	47–54	54.7 ± 4.51
NO_2_^−^-N	(mg N/L)	13.4 ± 4.15	34–56	52.6 ± 1.42
TN	(mg N/L)	3450 ± 500	3600–4800	3810 ± 161
N_org_	(mg N/L)	1680 ± 773	2129–3240	2300 ± 219
PO_4_^3-^-P	(mg P/L)	281 ± 153	15–20	12.3 ± 3.53
pH	-	8.05 ± 0.250	8–8.3	7.78 ± 0.110

### 2.4 CH biochar and soil sorption experiments

Nutrient sorption was measured in batch experiments with soil or CH biochar, and synthetic digestate solution. The detailed methodology and mass balance for the derivation of nutrient sorption coefficients is provided as SI.

### 2.5 Ammonia volatilization and leaching experiments

**Fig. 1:**
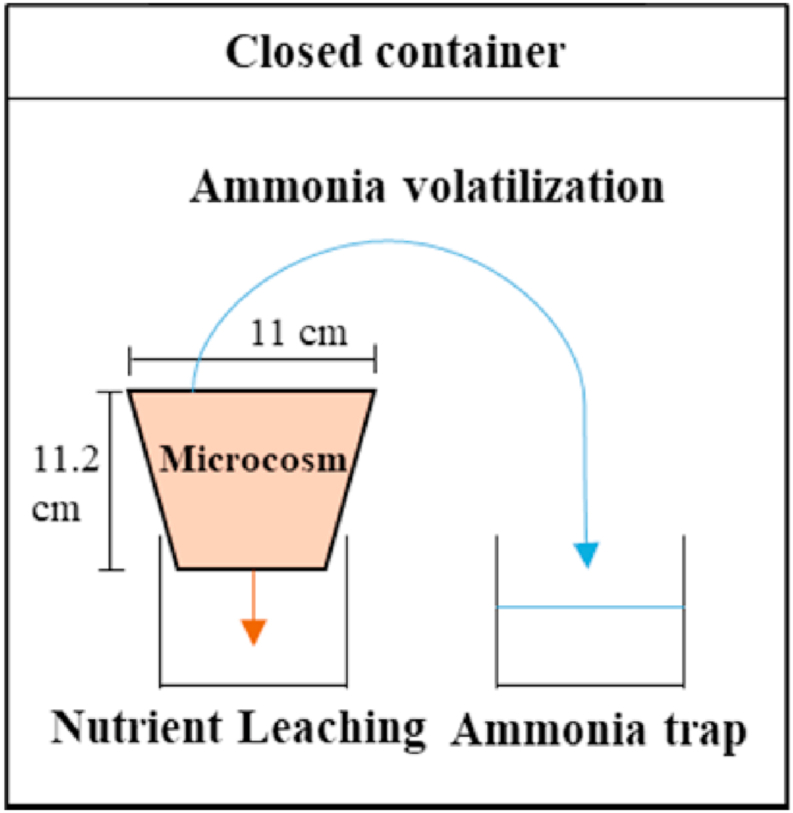
Schematic diagram of the sampling system for ammonia volatlization and nutrient leaching.

The experiments were conducted with soil microcosms placed over glass beakers for the leachate collection. Two soil microcosms with CH biochar (CH systems), and two soil microcosms without the biochar amendment (Control systems), were set up within closed polyethylene containers, which additionally contained an acidified distilled water trap for capturing gaseous ammonia during volatilization experiments (Fig. 1). Volatilization following digestate application to the CH and the Control systems was measured on days 1, 2, 11 and 28, and leaching following simulated heavy rainfall events was measured on days 7, 9, 16 and 30 to investigate CH biochar effects on nutrient losses from the digestate fertilized soil. Rainfall was simulated by adding 70 mL of distilled water to soil every 1 h for a 4-h period, based on universal high rainfall intensity at approximately 7 mm/h and the rate in Thailand at about 28–35 mm/day (TMD, 2016; Prakosa et al., 2018). A more detailed experimental methodology is provided in SI.

### 2.6 Molecular microbiology analysis

After 30 days, DNA was extracted from the top and bottom half of the soil microcosms and analyzed using 16S rRNA gene sequencing for microbial community characterization and qPCR of marker genes (amoA) to quantify microorganisms involved in the N cycle. The detailed methodology is provided as SI.

## 3 Results and discussion

### 3.1 CH biochar and soil sorption experiments

K_d_ measurements were performed to characterize the sorption of NH_4_^+^-N, NO_3_^−^-N, NO_2_^−^-N, TN, Urea-N_org_ and PO_4_^3-^-P by CH biochar and soil, and to estimate the CH amendment effect on nutrient sorption (Table 2). For NH_4_^+^-N, NO_3_^−^-N, TN and Urea-N_org_ the measurements supported the initial research hypothesis that soil amendment with biochar (K_d_, amended soil) could enhance nutrient retention. However, the anticipated impact, calculated using Eq.4 in SI, was small (Table 2). The biochar K_d_ values for NH_4_^+^-N, NO_3_^−^-N, TN and Urea-N_org_ showed similar and low K_d_ values, measuring 3.43 ± 0.99 L/kg, 1.90 ± 1.07 L/kg, 3.08 ± 1.66 L/kg and 2.99 ± 2.85 L/kg, respectively. Adsorption of NO_2_^−^-N, and PO_4_^3-^-P were too low for the derivation of a K_d_ value (SI, Table S6).

**Table 2:** Comparison of K_d_ (L/kg) of NH_4_^+^-N, NO_3_^−^-N, NO_2_^−^-N, TN, Urea-N_org_ and PO_4_^3-^-P in the biochar/soil-amended synthetic solution batch experiments and estimated K_d_ (L/kg) of the 10% (w/w) biochar amended soil. Results (Mean ± S.D.) are reported to two decimal places.

	NH_4_^+^-N	NO_3_^−^-N	NO_2_^−^-N	TN	Urea-N_org_	PO_4_^3-^-P
K_d_,_biochar_ (L/kg)	3.43 ± 0.99	1.90 ± 1.07	n/a	3.08 ± 1.66	2.99 ± 2.85	n/a
K_d_,_soil_ (L/kg)	0.80 ± 0.43	0.57 ± 1.38	0.56 ± 0.22	0.78 ± 0.50	0.78 ± 0.71	68.11 ± 20.40
K_d_,_amended soil_ (L/kg)	1.06 ± 0.40	0.70 ± 1.25	0.51 ± 0.19	1.01 ± 0.48	1.00 ± 0.71	61.30 ± 18.36

∗n/a = not available.

Biochar has heterogeneous surface properties with both, hydrophobic and hydrophilic characteristics, containing polar and non-polar surface sites which therefore can attract both polar and non-polar compounds (Hale et al., 2013; Ebrahimzadeh Omran et al., 2020). The adsorption of nutrients is normally controlled by the biochar surface chemistry (Lehmann and Joseph, 2015). NH_4_^+^-N adsorbed on CH biochar could be by electrostatic adsorption to negatively charged oxygen-containing surface functional groups, associated with cation exchange capacity (CEC) (Liu and Zhang, 2009; Tan et al., 2015). The CH biochar had H and O contents of 3.53% and 27.8%, respectively (SI, Section A1), implying the existence of hydroxyl (O-H) and other oxygen-containing functional groups such as C-O to form complexes on the biochar surface. These functional groups provided opportunity for cation, e.g. NH_4_^+^-N adsorption (Lui and Zhang, 2009).

CH biochar could also moderately adsorb NO_3_-^-^N. Although it was earlier explained that sorption to biochar is mainly governed by its CEC, anion exchange sites may coexist on the heterogeneous biochar surfaces. The condensed aromatic structures on the biochar are capable of generating positive surface charge, which presents some anion exchange capacity (AEC) (Lehmann and Joseph, 2015). Urea-N_org_ was also adsorbed by the biochar. Biochar contains both polar and non-polar surface sites which allows N_org_ attraction to both sites. Beesley et al. (2010) reported high adsorption of organic compounds to black carbon sorbents. However, urea is a small and polar organic molecule, which may explain its weak sorption to CH biochar.

Soil adsorbed all nutrients, however less than the biochar, except for PO_4_^3-^-P. A high K_d_ value was observed for PO_4_^3-^-P from soil. This is likely due to the soil physicochemical characteristics such as clay content, pH, and surface functional groups, e.g. Fe or Al oxides/hydroxides (Sparks, 2003). The adsorption of phosphate normally happens as inner-sphere complexes through a ligand exchange mechanism. The exchange is facilitated by elevating acidity and abundance of positive charges (Sparks, 2003). Additionally, Foth and Ellis (1996) reported that adsorption capacity of anions of monoprotic conjugate acids (a compound that can donate one proton) reaches a maximum when solution pH is close to the anion’s pK_a_. This agrees with the scenario for this experiment that the synthetic solution contained H_2_PO_4_^−^ with a pK_a_ of 7.2 and the solution pH was 7.66 (SI, Table S7).

### 3.2 Ammonia volatilization and nutrient leaching

**Fig. 2:**
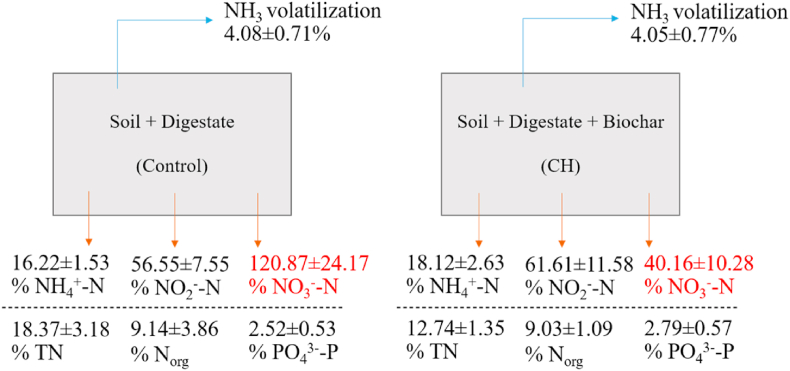
Total percentage of NH_4_^+^-N, NO_2_^−^-N, NO_3_^−^-N, TN, N_org_, PO_4_^3-^-P initially in the systems which was lost by leaching (orange arrows) and ammonia (NH_3_) volatilization (blue arrows) from digestate-amended soil (Control) and digestate-amended soil with CH biochar (CH) after four repeated volatilization and leaching experiments. Results (Mean ± S.D.) are reported to two decimal places. %NO_3_^−^-N was colored in red to emphasize the significant difference of the values between the two systems (*t*-test, p-value = 0.0007). (For interpretation of the references to color in this figure legend, the reader is referred to the Web version of this article.)

Fig. 2 shows the loss of each nutrient from the digestate-amended soil, and the digestate-amended soil with CH biochar, expressed as percentage of the amount of each nutrient initially present in each system. There was no significant difference between the ammonia volatilization from the CH and Control system (Fig. 2, *t*-test, p-value = 0.96), which was contrary to our hypothesis. This could be because the biochar did not significantly alter NH_4_^+^-N sorption (Table 2), therefore had little impact on volatilization. Also, ammonia mainly volatilized from the digestate on the soil surface which had not had opportunity to interact with the soil or biochar amended soil, hence no difference could be noticed in the volatilization rates. Similarly, Sha et al. (2019) reported that on average, biochar addition to soil had no impact on ammonia volatilization. However, this varied with different soil, biochar and experimental conditions. Biochar applied to acidic soils following ammonium-based fertilizer could increase volatilization as a result of elevated soil pH and urea hydrolysis (Sha et al., 2019). In contrast, using wood-based or acidified biochar at appropriate rates could mitigate ammonia volatilization following application of poultry litter or urea N fertilizer (Doydora et al., 2011; Feng et al., 2017).

For leaching, among the six nutrient parameters in each system, the lowest percent nutrient loss was for PO_4_^3-^-P in both systems, due to high PO_4_^3-^-P sorption by the soil (Table 2). The highest percent nutrient loss via leaching was for NO_3_^−^-N in the Control system and NO_2_^−^-N in the CH system. TN showed the most significant loss in terms of absolute mass (SI, Fig. S2). When looking at the effect of biochar, there was only one significant difference between the CH and Control system for the parameter NO_3_^−^-N. Leached nitrate-N was significantly higher from the Control system at 120.87 ± 24.17% of the amount initially present as compared to 40.16 ± 10.28% in the CH system (*t*-test, p-value = 0.0007). Our initial hypothesis of reduced nutrient leaching from the biochar amended soil was thus confirmed for this parameter only. It is noteworthy that NO_3_^−^-N leaching in the Control system is more than 100% of the mass initially present in the system. This implies nitrate production which could be attributed to the nitrification of ammonia to nitrate. In most soils the nitrite produced by ammonia oxidizers does not accumulate but is quickly oxidized to nitrate by the nitrite-oxidizing bacteria, suggesting that complete nitrification can occur within a short period of time (Paul, 2007). Notably, NO_3_^−^-N in the CH system leached less than in the Control system which implies that CH biochar could retard nitrification in digestate-amended soil. N_org_ adsorption by the biochar could have reduced the rate of microbial N mineralization and hence the rate of NO_3_^−^-N leaching from the CH system (Laird et al., 2010). Yao et al. (2012) studied nutrient leaching in sandy soil using peanut hull and Brazilian pepperwood biochar and found that both biochars, pyrolysed at 600 °C, could reduce ammonium and nitrate leaching, while peanut hull biochar showed no phosphate sorption ability. These effects of peanut hull biochar on leaching were consistent with the present study results using CH biochar. Another study using poultry litter-amended soil with pinewood biochar also found that such amendments reduced ammonium and nitrate leaching from sandy loam soil (Bohara et al., 2019). In contrast, increased N leaching was also reported with biochar amendment associated with its application rate (Li et al., 2018). Enhanced net N mineralization was observed in soil amended with N fertilizer and manure biochar which could cause higher nitrate leaching (Yoo and Kang, 2012). To better understand the microbiology of the Control and CH systems, 16S rRNA gene amplicon sequencing was performed for DNA extracted from the top and bottom soil layers at the end of the experiments.

### 3.3 Cluster and PCA analysis of the overall microbial community in the soils

**Fig. 3:**
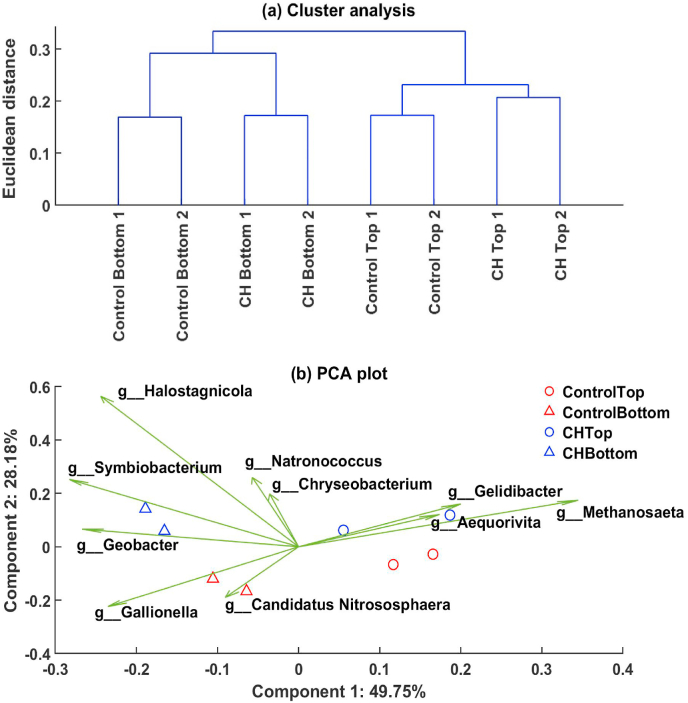
Cluster analysis (a) and Principal component analysis (PCA) (b) plots of the microbial community dissimilarity between the top and bottom soil samples of digestate-amended soil (Control) and digestate-amended soil with CH biochar (CH). For PCA, the first two principal components (Component 1 and 2) were plotted showing the scores (circles and triangles) and top 10 loadings (genera, arrows) in the two-dimensional space. Percentage of variation accounted for by each principal component is shown with the axis label.

Cluster analysis (Fig. 3a) shows the greatest dissimilarities for microbial communities were between samples from top and bottom soils, and then to a lesser extent in response to the biochar amendment. Sample replicates clustered most closely. One-way ANOSIM confirmed that top versus bottom soil was a significant factor in shaping the soil microbial communities (one-way ANOSIM, p-value < 0.05 and R = 0.80). In the principal component analysis (PCA, Fig. 3b), components 1 and 2 accounted for almost 78% of the observed variance between samples. Samples from top and bottom soils were separated along component 1, while component 2 separated the Control from the CH system samples. Evidently, the digestate application to the surface of the soils was the most significant microbial community shaping factor, while biochar amendment became influential in shaping the microbial community response to the digestate application within each soil layer.

The ten most notable microbial genera (i.e. variables) in the PC1 and 2 space are illustrated by the green arrows in Fig. 3b. These genera included *Candidatus Nitrososphera* and *Methanosaeta*, nitrifying archaea and methanogenic archaea, respectively. *Candidatus Nitrososphaera* was predominant in the Control bottom soil microbial community, while *Methanosaeta* was predominant in topsoil. The top 10 loadings for each PC 1 and 2 separately are presented in SI, Fig. S3. Clearly, the PCA highlighted nitrifying and methanogenic microbes as variables contributing to the sample dissimilarity showing that nitrifying and methanogenic microbes played an important role in the soil microbial community response to the digestate application. To confirm whether nitrification had likely occurred, and to study the biochar impacts on this process in more detail, the abundance of nitrifying microorganisms (AOB, AOA and NOB) was evaluated with a combination of sequencing and qPCR methods.

### 3.4 Abundance of nitrifying microorganisms

**Fig. 4:**
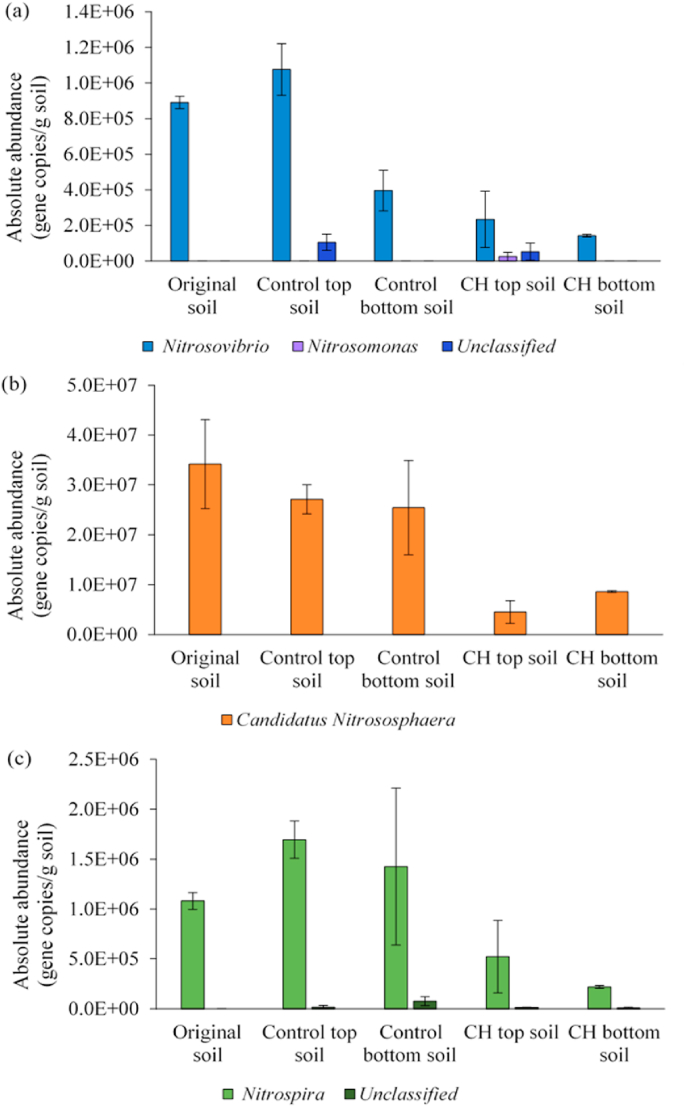
Genus abundance (gene copies/g. of soil) of AOB (a), AOA (b) and NOB (c) in original soil, digestate-amended soil (Control) and digestate-amended soil with CH biochar (CH). Data obtained from Illumina MiSeq 16S rRNA gene sequencing were combined with qPCR quantification of 16S rRNA gene copy numbers in each soil sample (see SI). Error bars calculated as standard deviation in duplicate CH and Control systems.

There were fewer nitrifying microorganisms overall in both the top and bottom soil of the CH system compared to the Control system (Fig. 4), and one genus of each nitrifier was driving the abundance differences between Control and CH systems. It can be seen from Fig. 4a that there was a lower mean AOB abundance in the CH system compared to the Control system. This demonstrates that the application of CH biochar with digestate led to the suppression of nitrifier populations, however, the difference was marginally not statistically significant (*t*-test, p-value = 0.09). The ammonia-oxidizing genus of *Nitrosovibrio* was predominantly detected. *Nitrosomonas* was only detected in CH topsoil, and an unclassified species of the *Nitrosomonadaceae* family was only detected in Control/CH topsoil, indicating that some nitrifiers might have been introduced with the biochar and/or digestate. The abundance of AOA (Fig. 4b) was generally more than one order of magnitude larger than the AOB in all soil samples. Overall, there was a significantly lower AOA abundance in the CH system compared to the Control system (*t*-test, p-value = 0.01). CH topsoil had significantly lower AOA abundance than Control topsoil (*t*-test, p-value = 0.03). Only the genus of *Candidatus Nitrososphaera* was found in both systems. Leininger et al. (2006) suggested that AOA are more numerous than AOB in soil, as was found in this study. The absolute abundance of NOB (Fig. 4c) was similar to that of AOB. Overall, the NOB abundance was significantly reduced in the system with CH biochar compared to the Control system and the original soil (*t*-test, p-value = 0.03 and 0.02, respectively). The *Nitrospira* genus was predominantly presented in both systems. There are alternative explanations for smaller nitrifier abundances in the CH system: (1) NH_4_^+^-N content is reduced through N_org_ immobilization by biochar, and the adsorption of NH_4_^+^-N as well as N_org_ by the biochar, which slows down N_org_ ammonification and ammonium availability for nitrification. Consequently, there is less NH_4_^+^ availability for oxidation by ammonia-consuming microbes and weakened nitrification in the soil (Wang et al., 2015). (2) Leachable bio-oil compounds were formed during the biochar production, and released from the biochar into soil which may inhibit microbial activity and imped nitrification (Lee et al., 2003; Wang et al., 2013; Wang et al., 2015). Clough et al. (2010) reported that nitrification rates decreased by adding wood biochar in pasture soils, which was attributed to a nitrification-inhibiting compound (α-pinene), a condensate product on the fresh biochar. (3) Biochar amendment to soil can affect moisture contents, hydraulic properties and aeration in the soil (Novak et al., 2012; Barnes et al., 2014), which can all indirectly influence the fate of nutrients, soil microbiology and ultimately plant growth. The addition of biochar can increase or decrease soil water-holding capacity depending on biochar type and application rate as well as soil type (Devereux et al., 2012; Barnes et al., 2014), which will affect oxygen availability for nitrification. Complex soil-biochar-microbiota interactions may explain the variable literature reports of how biochar affects nitrification. Dempster et al. (2012) found that the rate of nitrification significantly decreased with *Eucalyptus marginata* biochar with either fertilizer N or compost amendment to soil, because of the limited substrate (NH_4_^+^-N) level in the presence of biochar in soil. However, Bi et al. (2017) found that soil nitrification was enhanced through the increased abundance of AOB in the combined application of rice straw biochar and nitrogen fertilizer like urea and (NH_4_)_2_SO_4_. Prommer et al. (2014) also reported that the AOB community increased with wood biochar amendment to arable soils thus accelerated nitrification. Xu et al. (2014) indicated that the AOB abundance had not been affected by the rice straw biochar pyrolysed at 500 °C amended to an acidic soil.

**Fig. 5:**
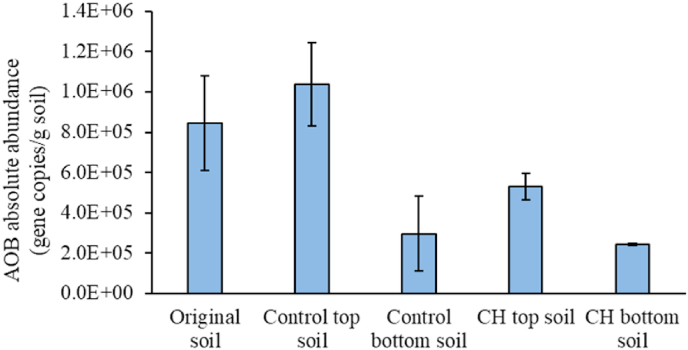
AOB absolute abundance (gene copies/g. of soil) obtained using amoA qPCR in original soil, digestate-amended soil (Control) and digestate-amended soil with CH biochar (CH). Error bars calculated as standard deviation in duplicate CH and Control systems.

Functional gene-specific qPCR (amoA) was also carried out (Fig. 5) to confirm the abundance results of AOB derived from 16S rRNA gene sequencing.

There was significantly lower amoA gene abundance in CH topsoil relative to Control topsoil (*t*-test, p-value = 0.002). The abundances of AOB in every samples obtained by amoA-based methods yielded abundance estimates very similar to those obtained from 16S rRNA gene sequencing using Illumina MiSeq (Fig. 4a). Song et al. (2014) conducted a study using qPCR of the amoA genes targeting AOA and AOB, and reported contrary results to those in this study. In their study, the abundance of both AOA and AOB increased in soil amended with cotton stalk biochar after four-week incubation and the AOB were more abundant than the AOA. Clearly, outcomes differ between studies, which may be attributed to variable biochar properties such as the characteristics of condensates formed from each biomass material under different pyrolysis conditions. A summary of the literature findings for different biochar types is provided in SI, Table S10. The summary shows that it is important to evaluate each biochar type separately before agricultural application.

### 3.5 Abundance of methanogens and methanotrophs

**Fig. 6:**
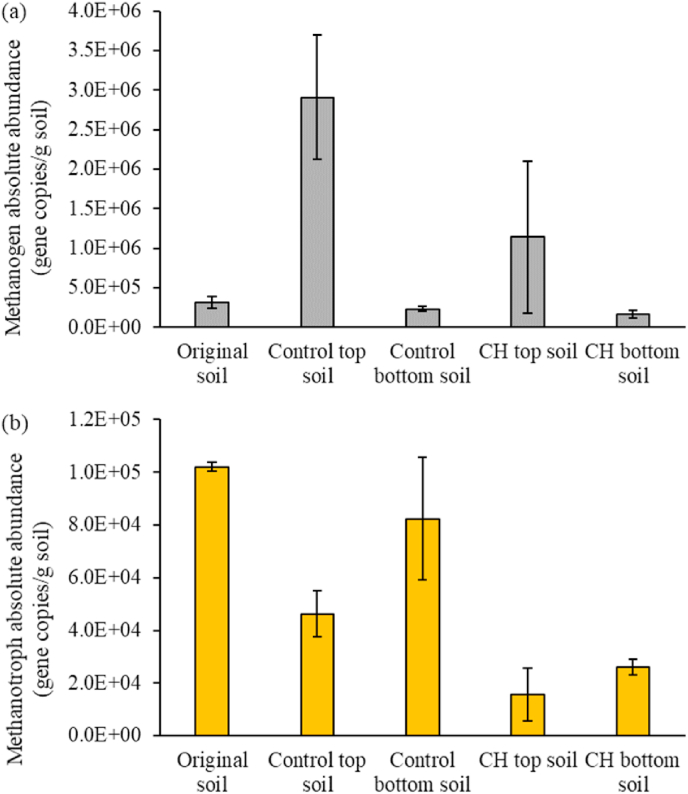
Abundance (gene copies/g. of soil) of methanogens (a) and methanotrophs (b) in original soil, digestate-amended soil (Control) and digestate-amended soil with CH biochar (CH). Data obtained from Illumina MiSeq 16S rRNA gene sequencing combined with qPCR quantification of 16S rRNA gene copy numbers in each soil sample (see SI). Error bars calculated as standard deviation in duplicate CH and Control systems.

Methanogens are anaerobic prokaryotes belonging to the domain Archaea, which are responsible for methane production (methanogensis) (Lew and Glińska-Lewczuk, 2018). Methanotrophs are microorganisms that oxidize methane as their sole carbon and energy source (methanotrophy) (Lew and Glińska-Lewczuk, 2018). Methanogenesis and methanotrophy take place simultaneously in the soil and such processes are associated with nitrification via ammonia oxidizers (Serrano-Silva et al., 2014). The enzyme MMO used for methanotrophy is capable of binding to NH_4_^+^ and react with it, and methanogens can use NH_4_^+^ as their N source (Serrano-Silva et al., 2014). Consequently, lower NH_4_^+^ substrate availability may reduce both methanotrophy and nitrification. Methanogens (genus *Methanosaeta*) were highlighted in the PCA of the overall microbial community (Fig. 3b). The abundance of methanogens and methanotrophs was therefore also analyzed in more detail (Fig. 6).

Fig. 6a shows a higher mean methanogen abundance in the topsoil than in the original soil and in the bottom soil of the Control system. However, the differences were marginally not statistically significant (*t*-test, p-value = 0.19 and 0.08, respectively). The application of digestate that contains methanogens to the soil surface can explain higher methanogen abundance in the topsoil layer. Additionally, most methanogens are able to function well in mesophilic environments (Garcia et al., 2000) and the digestate used in this study was obtained from a mesophilic digester. Similarly, it was reported that the top 7-cm soil layers were the primary methane production and diffusion sites, whilst the deeper soil layers acted as the sink (Xu et al., 2016b). However, even though the mean methanogen abundance was higher in the topsoil of the Control as compared to the CH system, there was no statistically significant difference (*t*-test, p-value = 0.39), indicating no impact of the biochar on methanogen abundances. Yuan et al. (2018) reported diverse biochar effects on methanogenesis. Wood chip biochar had little effect on methanogenesis in soil, whilst rice straw and manure biochar additions to soil enhanced methanogenesis remarkably due to the functional groups, mainly quinones, on the biochar surface. For methanotrophs (Fig. 6b), overall, there was lower methanotroph abundance in the CH system compared to the Control system (*t*-test, p-value = 0.05). Thus, CH biochar reduced methanotroph abundance, which could be attributed to the following reasons: (1) the organic compounds released from biochar that inhibited nitriﬁers also inhibited methanotrophs (Spokas, 2013). (2) Binding of organic compounds containing C/N from the digestate to the biochar led to less substrate availability for both methane and ammonium production, thus less substrate for methanotrophs and ammonium oxidizing bacteria, therefore less methanotroph and AOB abundances were detected in the CH systems. However, He et al. (2017) suggested that soils can also be more favorable for aerobic methanotrophs due to increased soil aeration by biochar addition. The addition of organic materials such as crop residues can diversely affect methanotrophic activity, depending on the C:N ratio of the materials (Serrano-Silva et al., 2014). Notably, methanotrophs could be found in both top and bottom soil of the Control system, but with slightly higher abundance in the bottom soil. Taipale et al. (2009) revealed that methanotrophs can adapt to microaerophilic conditions as well as anaerobic conditions. Moreover, Hu and Lu (2015) indicated that nitrate addition promoted the abundance and activity of methanotrophs in soil. In this present study, enhanced nitrate that was produced by the nitrifiers percolated down through the bottom soil, as was evident from the leaching results (Fig. 2). This may have promoted higher methanotroph abundance at the bottom. The abundance of methanotrophs in both Control and CH systems would likely be smaller than that in the original soil because of the applied digestate, since ammonium, methane oxidizers, as well as aerobic metabolizers of the digestate would compete for oxygen as an electron acceptor. Consequently, the addition of a rich substrate (digestate) would likely impede methanotroph abundance and thus methane oxidation (Serrano-Silva et al., 2014). Furthermore, CH biochar had higher pH than the soil (SI, Section 1, 2), thereby raising the soil pH. As methanotrophs are more sensitive to elevated soil pH than methanogens (Jeffery et al., 2016), CH biochar could have had a larger impact on the methanotrophs than the methanogens. A long-term study by Wang et al. (2019) reported that wheat straw biochar increased the abundances of both methanogens and methanotrophs in the first year of study, mainly due to enhanced in-soil dissolved organic carbon, NH_4_^+^-N, and porosity. However, after three years, the abundances of methanogens decreased.

## 4 Conclusions

This is the first study applying chemical measurements and molecular microbiology tools in combination to report the effects of the combined application of biochar and anaerobic digestate on ammonia volatilization, nutrient leaching and nitrification. CH biochar addition had no effect on ammonia volatilization, but reduced nitrate leaching by slowing down nitrification in digestate amended soil. There were lower nitrifying and methanotrophic microbe abundances in the biochar-amended soil following digestate application. CH biochar could thus ultimately retain nutrients longer for plant growth in the topsoil, reduce nitrate leaching during heavy rainfall events, and minimize groundwater pollution risks. The combined application of digestate with CH biochar is a promising biotechnology for sustainable agriculture, promoting the circular re-use of agricultural waste residues, in addition to renewable energy generation.

## Credit author statement

Jidapa Plaimart: Methodology, Investigation, Formal analysis, Data Curation, Writing - Original Draft, Visualization. Kishor Acharya: Investigation, Validation, Supervision, Writing - Review & Editing. Wojciech Mrozik: Supervision, Writing - Review & Editing. Russell J. Davenport: Supervision, Writing - Review & Editing. Soydoa Vinitnantharat: Resources, Writing - Review & Editing. David Werner: Conceptualization, Methodology, Software, Validation, Supervision, Writing - Review & Editing.

## Declaration of competing interest

The authors declare that they have no known competing financial interests or personal relationships that could have appeared to influence the work reported in this paper.
