# Supplementary material for: Coconut husk biochar amendment enhances nutrient retention by suppressing nitrification in agricultural soil following anaerobic digestate application
Source: Environ Pollut. 2021 Jan 1;268(Pt A):115684. doi: 10.1016/j.envpol.2020.115684 (PMC7762785; doi:10.1016/j.envpol.2020.115684)
Supplement: Multimedia component 1 [file mmc1.pdf]

Supplementary Information

Coconut husk biochar amendment enhances nutrient retention by suppressing nitrification in agricultural soil following anaerobic digestate application

Jidapa Plaimart[1], Kishor Acharya[1], Wojciech Mrozik[1], Russell J Davenport[1], Soydoa Vinitnantharat[2], David Werner[1]*

[1] School of Engineering, Newcastle University, Newcastle upon Tyne, NE1 7RU, United Kingdom

[2] Environmental Technology Program, School of Energy, Environment and Materials, King Mongkut's University of Technology Thonburi, Bangkok, 10140, Thailand

[*]Corresponding author, email address: david.werner@newcastle.ac.uk

A: Materials and Methods

A1. Biochar production

Biochar was produced by heating coconut husk in a 200 L oil drum kiln under oxygen limitation at the Center for Energy and Environmental Engineering, Kasetsart University, Kamphaeng Saen Campus, Thailand (Figure S1a). Prior to carbonization, coconut husk was air dried and weighed. To produce biochar in the oil drum kiln, a run was started by introducing 5 kg of dried coconut husks into the kiln. Then, the oven front was covered by clay, except for a square channel at the bottom front to feed in auxiliary fuels and allow some air and heat to circulate into the furnace. The size of the channel opening at the front was 20 x 20 cm^2 (Figure S1a). The process of carbonization started with the burning of auxiliary fuel, such as wood chips or firewood, in the front channel of the kiln. The heat from the burning fuel then flowed into the kiln. The heat first evaporated residual moisture in the coconut husk, which would take two to three hours. When the carbonization occurred, it could be noticed by the smoke released from the chimney. If there was a lot of white smoke released, it indicated that the coconut husks were heated and starting to partially combust. Air intake was then reduced by covering the front channel to about one-fourth. The carbonization proceeded continuously via circulated heat inside the kiln. Once the smoke from the chimney was lessened and became clear smoke, or if there was only hot air released from the chimney, this showed that coconut husks were becoming biochar. Clay was then used to completely cover the front channel of the kiln and other leaks in order to prevent outside air to pass through the

kiln. The kiln was left for about 1 night to complete the carbonization. The biochar was harvested once the kiln was cooled. The run was repeated for three times. A biochar yield of 33.7% was obtained from each run, and thus a total of 5 kg biochar was obtained from the three runs. For one run, the temperature during biomass conversion to biochar of the first run was measured every 10 minutes using a thermocouple inside the kiln. The maximum chamber temperature of 378 °C was measured at 70 minutes (Figure S1b). The biochar elemental composition included 68.4% C, 3.53% H, 27.8% O, 0.06% N and 0.15% S, and its pH was 9.8. The biochar BET surface area was 11 m^2/g and total porosity was 0.92. A well-homogenized, composite biochar sample was used for the experiments. The biochar was ground using a mortar and pestle and then sieved, and the <212 μm particle size fraction was used for the experiments.

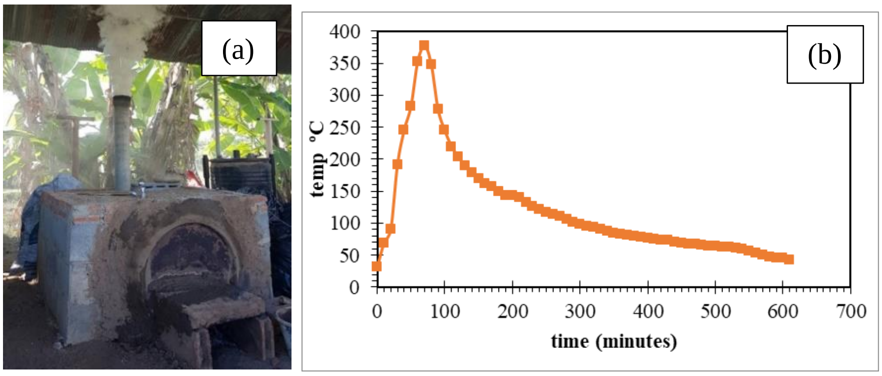

Figure S1. Oil drum kiln (a) and the chamber temperature profile (b).

A2. Sampling of soil and digestate

Clay loam soil with a pH of 6.4 was collected from arable land at approximately 0-10 cm depth. The digestate was collected from a 650 m^3 mesophilic anaerobic digester (41 °C), which was digesting mainly slurries of pig and cattle manure, which was sometimes augmented with energy crop and food residues. The digester was a continuous stirred-tank reactor (CSTR), in which feedstock materials were fed in every hour at a rate of approximately 0.4 m^3 per hour.

A3. Digestate characterization

For the real digestate used in this study, pH was measured using a Jenway pH Meter 3310. Nutrient characteristics including total nitrogen (TN), ammonium (NH_4^+-N), nitrate (NO_3^--N), nitrite (NO_2^--N) and phosphate (PO_4^{3-}-P) were determined from 1:100 distilled water diluted digestate using cuvette tests LCK338, LCK302, LCK340, LCK341, and LCK350, from HACH LANGE (Laser House, Manchester, M50 3XW, UK), respectively. Tests were

performed following the manufacturer’s instructions and measured in a HACH DR6000 Ultraviolet and Visible Spectrum Spectrophotometer. Organic nitrogen (N_{org}) was calculated according to $N_{org} = TN - (NH_4^+ - N + NO_3^- - N + NO_2^- - N)$. Then, a synthetic digestate solution was also prepared (Table S1), and its nutrient compositions and pH were measured directly from the solution following the same procedures as for real digestate.

Table S1. Target concentration for each nutrient parameter and its chemical concentration in the synthetic digestate solution.

Parameter	Target concentration (mg/L)	Chemical	Molecular weight (g/mol)	Chemical concentration (mg/L)	Chemical concentration (g/L)
NH_4^+-N (mg N/L)	1400	NH_4Cl	53.49	5349.10	5.35
NO_3^--N (mg N/L)	50	$NaNO_3$	85.00	303.55	0.30
NO_2^--N (mg N/L)	50	$NaNO_2$	69.00	246.41	0.25
TN (mg N/L)	3800	Urea	60.06	4933.5*	4.93
PO_4^{3-}-P (mg P/L)	15	Na_2HPO_4	141.96	68.69	0.07

* Urea concentration for TN was calculated from the target concentration of TN-(NH_4^+-N+NO_3^--N+NO_2^--N): 3800-(1400+50+50) = 2300 mg/L.

A4. CH biochar and soil sorption experiments

Sorption experiments were conducted using the synthetic digestate solution, which was initially prepared with $NaNO_3$, $NaNO_2$ and Na_2HPO_4. The solution was then autoclaved in a Rodwell autoclave at 121 °C for 15 minutes to minimize the potential of biodegradation in the sorption batch tests. NH_4Cl and urea, which were found to be unstable in the autoclaving process, were added to the autoclaved solution. The solution pH was also adjusted to mimic typical digestate pH around 8 using NaOH 1 N at 0.91 mL. The pH was measured using a Jenway pH Meter 3310. The concentration of each nutrient parameter in the synthetic digestate solution was also measured again after preparation (Table S6) by HACH LCK cuvette tests, as detailed in Section A3. The synthetic solution was used for sorption experiments promptly after the addition of urea and NH_4Cl. The soil sample was sterilized in an oven at 104 °C for 20 hours. 1.5 g of CH biochar or soil was mixed with 30 mL of the synthetic digestate solution. The pH of each mixture was measured before placing them on a Stuart Orbital Shaker SSLI running at 101 RPM for 16 hours. At the end of 16 hours, the pH of each mixture was measured again. Each sample was then filtered using a sterile syringe and 0.2 μm syringe filter. The batch experiment was performed in duplicate and with two sets of controls. Control A contained only 30 mL the synthetic digestate solution and Control B contained 1.5 g of CH biochar or soil with 30 mL of distilled water to measure the nutrient

release from the biochar or soil. The same procedures as in Section A3 were followed for nutrient measurement from the filtrates, but no dilution was necessary for Control B.

A5. Sorption coefficient modelling

The concentration of each nutrient parameter from the biochar/soil sorption experiment was used to calculate a linear sorption coefficient (K_d) (Eq.1). The derivation of the K_d equation, which considered in a mass balance both, the amount of nutrient associated with the biochar/soil and added to the batches as synthetic digestate, is also shown below.

$$K_d = \frac{(C_i \times V_w + C_{eq,cont} \times V_w - C_{eq} \times V_w)}{m \times (C_{eq} - C_{eq,cont})} \quad \text{(Eq.1)}$$

Where: K_d = Sorption coefficient (L/kg), C_i = Initial concentration of nutrient in solution (mg/L), V_w = Volume of solution (L), $C_{eq,cont}$ = Equilibrium concentration of the nutrient in control batches with the sorbent and distilled water (mg/L), m = Mass of adsorbent (kg) and C_{eq} = Equilibrium (final) concentration of the nutrient in batches with the sorbent and synthetic digestate solution (mg/L).

<u>K_d equation derivation</u>

Control batches with distilled water and biochar or soil:

$$V_w \times C_{eq,cont} + m \times K_d \times C_{eq,cont} = M_{nutrient,solid} \quad \text{(Eq.2)}$$

Where: $M_{nutrient,solid}$ (mg) is the amount of nutrient initially associated with the biochar or soil.

Batches with synthetic digestate solution and biochar or soil:

$$C_{eq} (V_w + m \times K_d) = V_w \times C_i + M_{nutrient,solid} \quad \text{(Eq.3)}$$

Substitute (Eq.2) in (Eq.3).

$$C_{eq} (V_w + m \times K_d) = V_w \times C_i + V_w \times C_{eq,cont} + m \times K_d \times C_{eq,cont}$$

$$C_{eq} \times V_w + C_{eq} \times m \times K_d = V_w \times C_i + V_w \times C_{eq,cont} + m \times K_d \times C_{eq,cont}$$

$$C_{eq} \times m \times K_d - m \times K_d \times C_{eq,cont} = V_w \times C_i + V_w \times C_{eq,cont} - C_{eq} \times V_w$$

$$m \times K_d (C_{eq} - C_{eq,cont}) = V_w \times C_i + V_w \times C_{eq,cont} - C_{eq} \times V_w$$

$$K_d = \frac{(C_i \times V_w + C_{eq,cont} \times V_w - C_{eq} \times V_w)}{m \times (C_{eq} - C_{eq,cont})}$$

A6. Ammonia volatilization and leaching experiments

Experiments were set up in duplicate including digestate-amended soil with CH biochar (2 x CH system) and without biochar (2 x Control system). The CH system contained 300 g of soil homogeneously mixed with 30 g of CH biochar and 30 mL of digestate was then applied on the soil surface based on a recommended application rate of 30 m^3/ha (Clarke, 2018). The Control system contained 300 g of soil with 30 mL of digestate applied on top in clay flowerpots. These pots had a hole for water drainage by gravity at the bottom and were placed on a glass beaker to collect leachate. They were enclosed within polyethylene containers closed with a lid. Two types of experiments were conducted:

(a) Ammonia volatilization experiment: to trap ammonia (NH_3) lost by volatilization from the soil as ammonium, a liquid trap containing 100 mL of distilled water acidified with 0.54 µL of 0.1 N HCl to a pH value of 3 was added to the enclosed systems. The ammonium in the trap was measured using cuvette test LCK302 every 2 hours for a 6-hour period, except for the first and last experiments which were monitored longer (see Table S8)

(b) Leaching experiment: rainfall was simulated by adding 70 mL of distilled water to soil every 1 hour for a 4-hour period. Leachate freely draining from the flowerpots into beakers was then collected to be analyzed for volume, pH and nutrient concentrations (TN, NH_4^+-N, NO_3^--N, NO_2^--N, PO_4^{3-}-P and N_{org}) using the same procedure for pH and nutrient measurement as detailed in Section A3.

Ammonia volatilization and leaching experiments were carried out over a period of one month using the same Control and CH microcosms with ammonia volatilization experiments carried out on day 1, 2, 11 and 28, while nutrient leaching experiments were conducted on day 7, 9, 16 and 30.

A7. Mass balance

The total amounts of each nutrient parameter initially in the CH and Control systems were calculated from the sum of the mass of each nutrient species (TN, NH_4^+-N, NO_3^--N, NO_2^--N, PO_4^{3-}-P and N_{org}) in soil, CH biochar and digestate at the beginning of the experiments in order to account for the ammonia volatilization and nutrient leaching data as a percentage of the total nutrient mass initially present in the system. Results of the total mass of each nutrient in each system can be seen from Table S2. Moreover, because of the nutrient content of the biochar, it can be noticed that the amounts of nutrients in the CH system were initially up to 19.6% higher than those for the Control system.

Table S2. Total mass (mg) of NH_4^+-N, NO_2^--N, NO_3^--N, TN, N_{org} and PO_4^{3-}-P in the Control and CH system. Results (Mean±S.D.) are reported to two decimal places.

	NH_4^+-N (mg)	NO_2^--N (mg)	NO_3^--N (mg)	TN (mg)	N_{org} (mg)	PO_4^{3-}-P (mg)
Control system	58.03±8.93	1.79±0.20	41.52±12.46	555.48±20.72	453.72±30.54	137.29±32.70
CH system	59.48±8.93	2.14±0.20	46.87±12.54	567.70±20.76	458.44±30.63	152.58±32.89

A8. Molecular microbiology analysis

A8.1 DNA extraction, quantification and 16S rRNA gene sequencing

The total DNA from the biomass contained in the top and bottom soils was extracted using the FASTDNA Spin Kit for soil according to the manufacturer's instructions (MPBiomedicals, Santa Ana, CA, USA). The concentration of total DNA extracted was determined using a Qubit® dsDNA HS Assay Kit (Invitrogen, Life Technologies, Paisley, UK). Finally, the extracted DNA was stored at -20 ^oC until further use. Total extracted DNA was submitted for sequencing (paired end sequencing; 2 × 250 bp) in duplicate with an Illumina Miseq platform at the Department of Applied Biology, Cellular and Molecular Sciences, Northumbria University, UK using the primer set targeting the V4 region of the prokaryotic16S rRNA gene, as described elsewhere (Kozich *et al.*, 2013; Acharya *et al.*, 2019).

A8.2 Bioinformatic analysis

The amplicon sequencing data from Illumina were processed using an open source software package; Quantitative Insight Into Microbial Ecology, QIIME 2 (http://www.qiime.org). Denoising and de-replication of pair end sequencing, including chimera removal and trimming of reads based on positional quality scores, were performed using the Divisive Amplicon Denoising Algorithm 2 (DADA2) (Callahan *et al.*, 2016). The quality filtered sequences were clustered into ASVs (amplicon sequencing variants) by using the VSEARCH clustering method, which were then converted into OTUs (operation taxonomic units), with a threshold of 97% identity (Rognes *et al.*, 2016). Finally, the taxonomy for each OTU was assigned by matching against the GreenGenes database (v13_8), based on a naïve Bayesian classifier with default parameters. Sequencing libraries were rarefied at 45,000 reads per sample, and multivariate statistical analysis was performed for OTUs classified to genus level, and grouped at this level, using Matlab © for cluster and principal component analysis with Euclidean distance as the similarity metric. ANOSIM was performed with the Fathom

Toolbox for Matlab© developed by the Marine Resource Assessment Program at the University of South Florida’s College of Marine Science (Jones, 2015).

A8.3 qPCR on marker genes

The number of target genes were quantified by Real time PCR assays (qPCR) on a BioRad CFX C1000 system (BioRad, Hercules, CA USA) using the primers shown in Table S3. Regarding quantification of the target genes (i.e. 16S rRNA gene and amoA gene from AOB), DNA samples were firstly diluted to a working solution of 10 ng/uL to prevent inhibitor effects, 2 μL template DNA was then used in a reaction mixture containing 7.5 μL 2 × SsoAdvanced Universal SYBR Green Supermix (Bio-Rad), 500 nmol L^{-1} of each forward and reverse primer (Table S3), and molecular grade H_2O (Invitrogen, Life Technologies, Paisley, UK) to a final volume of 15 μL. Reaction conditions for quantification of each target gene were 98 °C for 3 minutes (1x), then 98 °C for 15 seconds, and the Primer Annealing Temperature (T_a) for 60 seconds (Table S3) (40 cycles). Standard curves were formed using the synthesized nucleotide sequence of the target gene (Invitrogen, Life Technologies, Paisley, UK), and produced every time a qPCR analysis was performed, in parallel with the amplification of test samples. Serial dilution (10-fold) of the standards was performed to obtain standard solutions in the range of 10^8–10^1 target gene copies/μL. All samples were run in duplicate and molecular grade H_2O replaced template in control reactions.

For sequencing data processing, in order to calculate absolute abundance of the studied microorganisms, the accurate number of 16S rRNA genes per genome of each microorganism was required. The data were obtained from the ribosomal RNA operon copy number database (http: //rrndb.umms.med.umich.edu/) with the following details.
AOB, AOA, NOB: 1 copy of 16S/genome**,** Methanogens: 3 copies of 16S/genome
Methanotrophs: 2 copies 16S/genome.

Table S3. Real-time qPCR primers for different genetic markers.

Target organisms	Primer	Sequence (5'>>>3')	Annealing Temperature (T_a)	Amplicon size	Reference
Total Bacteria (16S rRNA); qPCR	1055 F	ATGGCTGTCGTCAGCT	60 ºC	337	Harms *et al.* (2003)
	1392 R	ACGGGCGGTGTGTAC			
AOB	amoA-1F*	GGGGHTTYTACTGGTGGT	56 ºC	490	Stephen *et al.* (1999)
	amoA-2R	CCCCTCKGSAAAGCCTTCTTC			
16S rRNA gene sequencing; Illumina	515 F	GTGCCAGCMGCCGCGGTAA	55 ºC	291	Kozich *et al.* (2013)
	806 R	TAATCTWTGGGVHCATCAGG			

A9. Statistical analysis

Two-tailed statistical T-Test was performed at significance level $\alpha = 0.05$ ($p\text{-value} \leq 0.05$) to predict significant differences in nutrient losses from ammonia volatilization and leaching, also the differences in the abundances of nitrifying and methane-producing/oxidizing microbes between Control and CH systems. One-way ANOSIM was used to predict the P-value and R-value of ranked dissimilarities between versus within groups of samples.

B: Additional results

B1. To estimate K_d of the biochar amended soil (L/kg)

$$K_{d,amended\ soil} = (1 - f_{bc}) \times K_{d,soil} + f_{bc} \times K_{d,biochar} \quad \text{(Eq.4)}$$

f_{bc} is the weight fraction of the biochar in the soil, K_{d}, soil (L/kg) is the soil K_d of each nutrient and $K_{d,biochar}$ (L/kg) is the biochar K_d of each nutrient

f_{bc} in this case is 0.09 calculated from the ratio of biochar to total weight in soil (30 g: 330 g)

$$K_{d,amended\ soil} = (1 - 0.09) \times K_{d,soil} + 0.09 \times K_{d,biochar} \quad \text{(Eq.5)}$$

B2. Ammonia volatilization and nutrient leaching

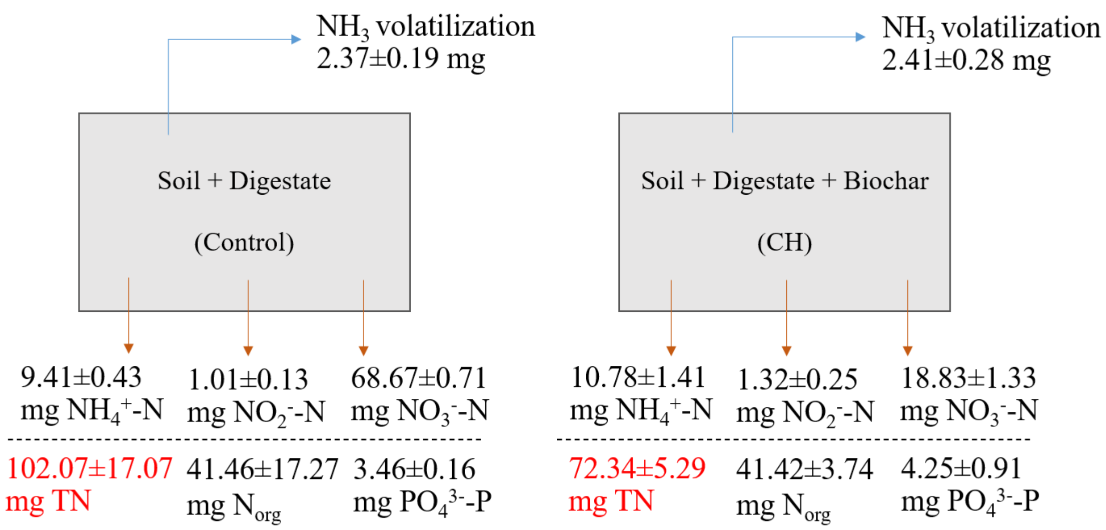

Figure S2. Total mass (mg) of NH_4^+-N, NO_2^--N, NO_3^--N, TN, N_{org}, PO_4^{3-}-P which was lost by leaching (orange arrows) and ammonia (NH_3) volatilization (blue arrows) from digestate-amended soil (Control) and digestate-amended soil with CH biochar (CH) after four repeated volatilization and leaching experiments. Results (Mean±S.D.) are reported to two decimal places. mg TN was colored in red to emphasize the highest loss in terms of absolute mass among all nutrients in both systems.

B3. PCA analysis

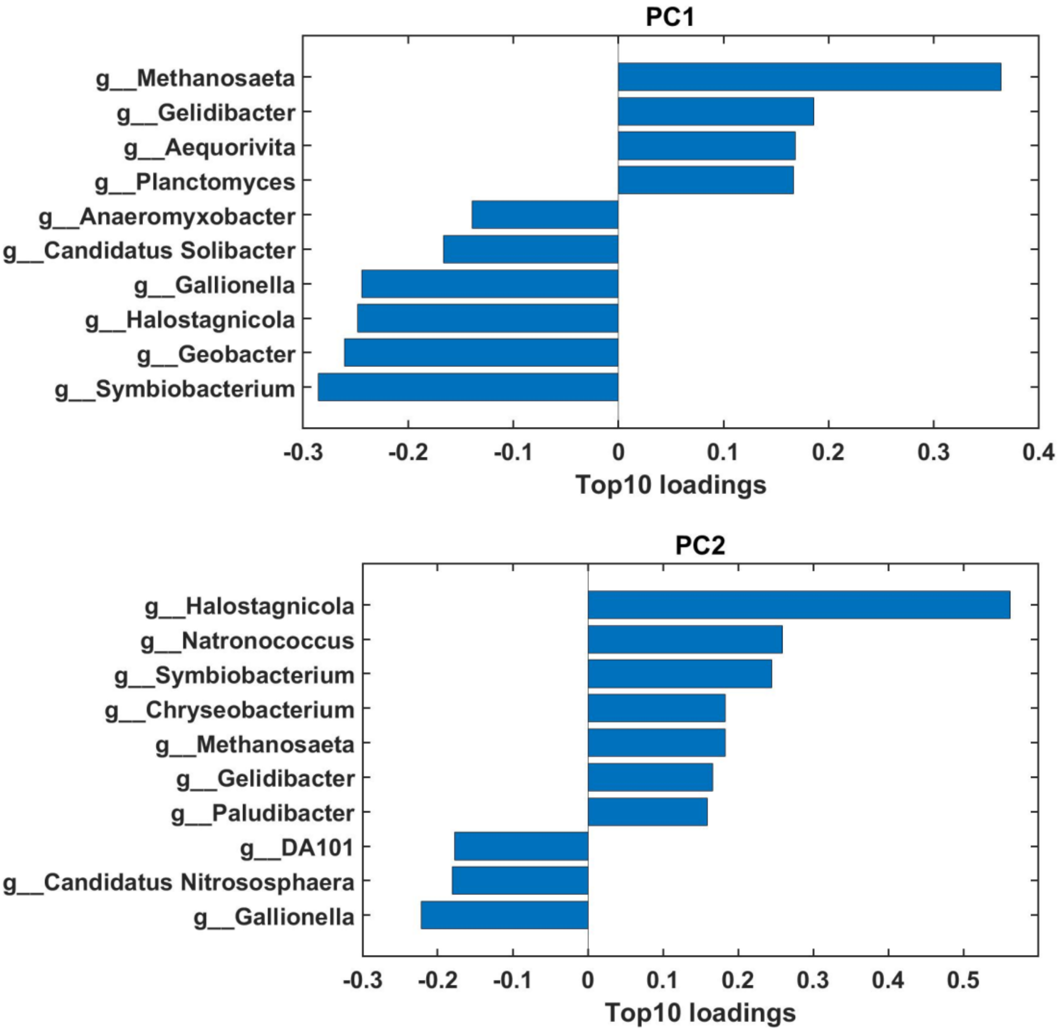

Figure S3. Variables (microbial genera) with the top 10 loadings for component 1 (PC1) and component 2 (PC2), respectively, of the principal component analysis (PCA).

B4. DNA Sequencing and qPCR

Table S4. Relative abundances of different nitrifying microbes, methanogens and methanotrophs estimated from 16S rRNA genes sequenced with Illumina MiSeq sequencing in the original soil, top and bottom soil samples of digestate-amended soil (Control) and digestate-amended soil with CH biochar (CH), and their replicates. n.d. indicates not detected.

	AOB			AOA	NOB		Methanogens	Methanotrophs
Sample	*Nitrosovibrio*	*Nitrosomonas*	Unclassified	*Candidatus Nitrososphaera*	*Nitrospira*	Unclassified		
Original soil 1	1.39E-03	n.d.	n.d.	6.50E-02	1.76E-03	n.d.	1.74E-03	3.03E-04
Original soil 2	1.81E-03	n.d.	n.d.	5.34E-02	2.11E-03	n.d.	1.53E-03	4.39E-04
Control topsoil 1	1.77E-03	n.d.	2.89E-04	4.61E-02	2.87E-03	n.d.	2.11E-02	2.10E-04
Control topsoil 2	2.75E-03	n.d.	1.36E-04	6.75E-02	4.24E-03	7.78E-05	1.43E-02	1.69E-04
Control bottom soil 1	1.05E-03	n.d.	n.d.	7.20E-02	4.57E-03	2.53E-04	1.60E-03	4.36E-04
Control bottom soil 2	1.07E-03	n.d.	n.d.	6.08E-02	2.43E-03	1.21E-04	2.33E-03	4.50E-04
CH topsoil 1	1.86E-03	2.35E-04	4.79E-04	3.20E-02	4.19E-03	8.24E-05	2.98E-02	2.43E-04
CH topsoil 2	1.68E-03	4.70E-05	1.10E-04	5.01E-02	3.55E-03	2.35E-04	1.21E-02	2.51E-04
CH bottom soil 1	9.21E-04	n.d.	n.d.	5.72E-02	1.59E-03	1.21E-04	2.45E-03	3.96E-04
CH bottom soil 2	9.01E-04	n.d.	n.d.	5.27E-02	1.22E-03	n.d.	3.78E-03	2.78E-04

Table S5. 16S rRNA gene copy numbers obtained from qPCR quantification in the original soil, top and bottom soil samples of digestate-amended soil (Control) and digestate-amended soil with CH biochar (CH), and their replicates.

Sample	gene copies/g soil
Original soil 1	6.63E+08
Original soil 2	4.73E+08
Control topsoil 1	5.25E+08
Control topsoil 2	4.44E+08
Control bottom soil 1	4.84E+08
Control bottom soil 2	2.63E+08
CH topsoil 1	2.12E+08
CH topsoil 2	4.50E+07
CH bottom soil 1	1.47E+08
CH bottom soil 2	1.67E+08

C: Raw data

Table S6. pH and nutrient concentrations (C_{eq}) of NH_4^+-N, NO_3^--N, NO_2^--N, TN, PO_4^{3-}-P and N_{org} (mg/L) in batch experiments containing biochar-amended synthetic solutions and two sets of control. Results are reported to two decimal places.

Sample	Biochar (g)	pH pre contact time	pH post contact time	NH_4^+-N (mg N/L)	NO_3^--N (mg N/L)	NO_2^--N (mg N/L)	TN (mg N/L)	PO_4^{3-}-P (mg P/L)	N_{org}* (mg N/L)
CH 1	1.5	8.51	8.21	1120.00	55.00	52.20	3690.00	39.40	2462.80
CH 2	1.5	8.54	8.18	1180.00	53.75	53.00	3210.00	42.00	1923.25
Control A1	X	7.88	7.81	1300.00	50.23	50.50	3890.00	8.60	2489.27
Control A2	X	7.88	7.76	1390.00	51.00	52.10	4030.00	9.00	2536.90
Control B1	1.5	9.78	9.75	2.13	10.30	0.60	16.50	32.20	3.47
Control B2	1.5	9.76	9.71	2.01	6.00	0.60	18.80	28.30	10.19

* N_{org} data was obtained from calculation

CH = synthetic solution + 1.5 g biochar

Control A = synthetic solution only

Control B = distilled water + 1.5 g biochar

Table S7. pH and nutrient concentrations (C_{eq}) of NH_4^+-N, NO_3^--N, NO_2^--N, TN, PO_4^{3-}-P and N_{org} (mg/L) in batch experiments containing soil-amended synthetic solutions and two sets of control. Results are reported to two decimal places.

Sample	Soil (g)	pH pre contact time	pH post contact time	NH_4^+-N (mg N/L)	NO_3^--N (mg N/L)	NO_2^--N (mg N/L)	TN (mg N/L)	PO_4^{3-}-P (mg P/L)	N_{org}* (mg N/L)
Soil 1	1.5	7.45	6.89	1420.00	65.00	52.80	3670.00	7.80	2132.20
Soil 2	1.5	7.37	6.82	1410.00	61.30	52.30	3520.00	9.10	1996.40
Control A 1	X	7.67	7.66	1440.00	61.50	54.30	3620.00	14.90	2064.20
Control A 2	X	7.67	7.66	1500.00	55.90	53.30	3700.00	16.60	2090.80
Control B 1	1.5	6.40	6.23	1.43	8.05	0.20	74.00	5.35	64.32
Control B 2	1.5	6.43	6.25	1.52	4.10	0.25	71.00	4.40	65.13

* N_{org} data was obtained from calculation

Soil = synthetic solution + 1.5 g soil

Control A = synthetic solution only

Control B = distilled water + 1.5 g soil

Table S8. NH_4^+-N (mg) in the acidic water trap as a function of time of the system containing digestate-amended soil with CH biochar (CH) and digestate-amended soil only (Control) on day 1, 2, 11 and 28. Results are reported to three decimal places.

Day	Hour	NH_4^+-N (mg)			
		CH 1	CH 2	Control 1	Control 2
1	0	0.000	0.000	0.000	0.000
	2	0.111	0.270	0.129	0.132
	4	0.318	0.586	0.308	0.297
	17	1.770	2.330	1.860	2.170
2	0	0.000	0.000	0.000	0.000
	2	0.073	0.081	0.066	0.106
	4	0.186	0.194	0.168	0.218
	6	0.287	0.279	0.290	0.338
11	0	0.000	0.000	0.000	0.000
	2	0.010	0.007	0.000	0.000
	4	0.047	0.045	0.017	0.027
	6	0.076	0.075	0.030	0.045
28	0	0.000	0.000	0.000	0.000
	2	0.002	0.000	0.000	0.000
	5	0.000	0.000	0.000	0.000
	8	0.000	0.000	0.000	0.000

Table S9. pH and mass of each nutrient (NH_4^+-N, NO_3^--N, NO_2^--N, TN, PO_4^{3-}-P and N_{org}) (mg) lost by leaching from the system containing digestate-amended soil with CH biochar (CH) and digestate-amended soil only (Control) on day 7, 9, 16 and 30. Results are reported to two decimal places.

Day	System	pH	NH_4^+-N (mg)	NO_3^--N (mg)	NO_2^--N (mg)	TN (mg)	PO_4^{3-}-P (mg)	N_{org}* (mg)
7	Control 1	6.11	2.82	31.20	0.10	77.04	0.66	42.93
	Control 2	5.75	3.16	31.27	0.11	42.93	0.62	8.40
	CH 1	7.45	3.83	12.57	0.08	40.92	0.38	24.43
	CH 2	7.52	3.19	14.25	0.06	36.23	0.29	18.72
9	Control 1	6.83	2.68	8.40	0.63	20.58	1.05	8.87
	Control 2	6.55	3.38	8.29	0.42	21.86	1.23	9.76
	CH 1	7.93	4.82	2.40	1.10	19.68	2.66	11.35
	CH 2	8.11	3.02	1.17	0.62	11.48	1.27	6.67
16	Control 1	7.50	2.90	7.30	0.29	16.63	1.15	6.14
	Control 2	7.11	3.11	7.51	0.21	17.33	1.40	6.50
	CH 1	7.98	3.65	2.82	0.28	17.01	2.06	10.26
	CH 2	8.15	1.89	1.18	0.28	12.51	1.03	9.17
30	Control 1	7.06	0.26	3.91	0.08	4.25	0.44	0.00
	Control 2	8.03	0.51	2.51	0.19	3.30	0.38	0.09
	CH 1	8.08	1.24	1.31	0.17	3.65	0.81	0.93
	CH 2	8.79	0.20	1.36	0.05	2.06	0.27	0.46

*N_{org} data was obtained from calculation

D: Literature comparisons

Table S10. Comparisons of biochar effects on ammonia volatilization, nutrient leaching and nitrification between this and literature studies.

	This study outcome	Studies with the same outcomes	Studies with different outcomes	Type of biochar used in each study
Ammonia volatilization	Coconut husk biochar had no significant effects on ammonia volatilization in soil	Sha *et al.* (2019)<br>Sun *et al.* (2017)	Reduced ammonia volatilization:<br>Doydora *et al.* (2011)<br>Mandal *et al.* (2016)<br>Taghizadeh-Toosi *et al.* (2012)<br><br>Increased ammonia volatilization:<br>Sun *et al.* (2014)<br>Chen *et al.* (2013)<br>Schomberg *et al.* (2012)	Sha *et al.* (2019): Ligno-cellulosic waste (Macadamia nutshell, walnut shells, peanut shells and maize cobs)<br>Sun *et al.* (2017): Wheat straw<br>Doydora *et al.* (2011): Wood<br>Mandal *et al.* (2016): Poultry litter and Macadamia nutshell<br>Taghizadeh-Toosi *et al.* (2012): Wood chips<br>Sun *et al.* (2014): Wheat straw<br>Chen *et al.* (2013): Green waste<br>Schomberg *et al.* (2012): Peanut hull
Nutrient leaching	Coconut husk biochar reduced nitrate leaching in soil	Yao *et al.* (2012)<br>Bohara *et al.* (2019)<br>Laird *et al.* (2010)<br>Zheng *et al.* (2013)	Increased nitrate leaching:<br>Li *et al.* (2018)<br>Yoo and Kang (2012)<br>Eykelbosh *et al.* (2015)	Yao *et al.* (2012): Peanut hull and Brazilian pepperwood<br>Bohara *et al.* (2019): Pinewood<br>Laird *et al.* (2010): Hardwood<br>Zheng *et al.* (2013): Giant reed<br>Li *et al.* (2018): Apple branches<br>Yoo and Kang (2012): Manure<br>Eykelbosh *et al.* (2015): Filtercake
Nitrification	Coconut husk biochar retarded nitrification in soil	Wang *et al.* (2015)<br>Clough *et al.* (2010)<br>Dempster *et al.* (2012)<br>Song *et al.* (2019)	Increased nitrification:<br>Bi *et al.* (2017)<br>Prommer *et al.* (2014)<br>He *et al.* (2019)<br>Zhao *et al.* (2020)	Wang *et al.* (2015): Peanut shell<br>Clough *et al.* (2010): Wood<br>Dempster *et al.* (2012): *Eucalyptus marginata*<br>Song *et al.* (2019): Bamboo leaf<br>Bi *et al.* (2017): Rice straw<br>Prommer *et al.* (2014): Wood<br>He *et al.* (2019): Rice straw<br>Zhao *et al.* (2020): Wheat straw

References

Acharya, K., Khanal, S., Pantha, K., Amatya, N., Davenport, R.J. and Werner, D. (2019) 'A comparative assessment of conventional and molecular methods, including MinION nanopore sequencing, for surveying water quality', *Scientific Reports*, 9(1), p. 15726.

Bi, Q.-F., Chen, Q.-H., Yang, X.-R., Li, H., Zheng, B.-X., Zhou, W.-W., Liu, X.-X., Dai, P.-B., Li, K.-J. and Lin, X.-Y. (2017) 'Effects of combined application of nitrogen fertilizer and biochar on the nitrification and ammonia oxidizers in an intensive vegetable soil', *AMB Express*, 7, p. 198.

Bohara, H., Dodla, S., Wang, J.J., Darapuneni, M., Acharya, B.S., Magdi, S. and Pavuluri, K. (2019) 'Influence of poultry litter and biochar on soil water dynamics and nutrient leaching from a very fine sandy loam soil', *Soil and Tillage Research*, 189, pp. 44-51.

Callahan, B.J., McMurdie, P.J., Rosen, M.J., Han, A.W., Johnson, A.J.A. and Holmes, S.P. (2016) 'DADA2: High-resolution sample inference from Illumina amplicon data', *Nature methods*, 13(7), pp. 581-583.

Chen, C.R., Phillips, I.R., Condron, L.M., Goloran, J., Xu, Z.H. and Chan, K.Y. (2013) 'Impacts of greenwaste biochar on ammonia volatilisation from bauxite processing residue sand', *Plant and Soil*, 367(1), pp. 301-312.

Clarke, A. (2018) *Q&A: Tips on using anaerobic digestate as a fertiliser*. Available at: https://www.fwi.co.uk/arable/crop-management/nutrition-and-fertiliser/qa-using-anaerobic-digestate-fertiliser (Accessed: 16 July 2019).

Clough, T.J., Bertram, J.E., Ray, J.L., Condron, L.M., O'Callaghan, M., Sherlock, R.R. and Wells, N. (2010) 'Unweathered wood biochar impact on nitrous oxide emissions from a bovine-urine-amended pasture soil', *Soil Science Society of America Journal*, 74(3), pp. 852-860.

Dempster, D.N., Gleeson, D.B., Solaiman, Z.M., Jones, D.L. and Murphy, D.V. (2012) 'Decreased soil microbial biomass and nitrogen mineralisation with Eucalyptus biochar addition to a coarse textured soil', *Plant and Soil*, 354(1), pp. 311-324.

Doydora, S.A., Cabrera, M.L., Das, K.C., Gaskin, J.W., Sonon, L.S. and Miller, W.P. (2011) 'Release of Nitrogen and Phosphorus from Poultry Litter Amended with Acidified Biochar', *International Journal of Environmental Research and Public Health*, 8(5), pp. 1491-1502.

Eykelbosh, A.J., Johnson, M.S. and Couto, E.G. (2015) 'Biochar decreases dissolved organic carbon but not nitrate leaching in relation to vinasse application in a Brazilian sugarcane soil', *Journal of Environmental Management*, 149, pp. 9-16.

Harms, G., Layton, A.C., Dionisi, H.M., Gregory, I.R., Garrett, V.M., Hawkins, S.A., Robinson, K.G. and Sayler, G.S. (2003) 'Real-Time PCR Quantification of Nitrifying Bacteria in a Municipal Wastewater Treatment Plant', *Environmental Science & Technology*, 37(2), pp. 343-351.

He, L., Shan, J., Zhao, X., Wang, S. and Yan, X. (2019) 'Variable responses of nitrification and denitrification in a paddy soil to long-term biochar amendment and short-term biochar addition', *Chemosphere*, 234, pp. 558-567.

Jones, D. (2015) 'Fathom Toolbox for Matlab: software for multivariate ecological and oceanographic data analysis', *College of Marine Science, University of South Florida, St. Petersburg, FL, USA.*

Kozich, J.J., Westcott, S.L., Baxter, N.T., Highlander, S.K. and Schloss, P.D. (2013) 'Development of a Dual-Index Sequencing Strategy and Curation Pipeline for Analyzing Amplicon Sequence Data on the MiSeq Illumina Sequencing Platform', *Applied and Environmental Microbiology*, 79(17), p. 5112.

Laird, D., Fleming, P., Wang, B., Horton, R. and Karlen, D. (2010) 'Biochar impact on nutrient leaching from a Midwestern agricultural soil', *Geoderma*, 158(3), pp. 436-442.

Li, S., Zhang, Y., Yan, W. and Shangguan, Z. (2018) 'Effect of biochar application method on nitrogen leaching and hydraulic conductivity in a silty clay soil', *Soil and Tillage Research*, 183, pp. 100-108.

Mandal, S., Thangarajan, R., Bolan, N.S., Sarkar, B., Khan, N., Ok, Y.S. and Naidu, R. (2016) 'Biochar-induced concomitant decrease in ammonia volatilization and increase in nitrogen use efficiency by wheat', *Chemosphere*, 142, pp. 120-127.

Prommer, J., Wanek, W., Hofhansl, F., Trojan, D., Offre, P., Urich, T., Schleper, C., Sassmann, S., Kitzler, B., Soja, G. and Hood-Nowotny, R.C. (2014) 'Biochar Decelerates Soil Organic Nitrogen Cycling but Stimulates Soil Nitrification in a Temperate Arable Field Trial', *PLOS ONE*, 9(1), p. e86388.

Rognes, T., Flouri, T., Nichols, B., Quince, C. and Mahé, F. (2016) 'VSEARCH: a versatile open source tool for metagenomics', *PeerJ*, 4, pp. e2584-e2584.

Schomberg, H.H., Gaskin, J.W., Harris, K., Das, K.C., Novak, J.M., Busscher, W.J., Watts, D.W., Woodroof, R.H., Lima, I.M., Ahmedna, M., Rehrah, D. and Xing, B. (2012) 'Influence of Biochar on Nitrogen Fractions in a Coastal Plain Soil', *Journal of Environmental Quality*, 41(4), pp. 1087-1095.

Sha, Z., Li, Q., Lv, T., Misselbrook, T. and Liu, X. (2019) 'Response of ammonia volatilization to biochar addition: A meta-analysis', *Science of The Total Environment*, 655, pp. 1387-1396.

Song, Y., Li, Y., Cai, Y., Fu, S., Luo, Y., Wang, H., Liang, C., Lin, Z., Hu, S., Li, Y. and Chang, S.X. (2019) 'Biochar decreases soil N2O emissions in Moso bamboo plantations through decreasing labile N concentrations, N-cycling enzyme activities and nitrification/denitrification rates', *Geoderma*, 348, pp. 135-145.

Stephen, J.R., Chang, Y.J., Macnaughton, S.J., Kowalchuk, G.A., Leung, K.T., Flemming, C.A. and White, D.C. (1999) 'Effect of toxic metals on indigenous soil beta-subgroup proteobacterium ammonia oxidizer community structure and protection against toxicity by inoculated metal-resistant bacteria', *Applied and environmental microbiology*, 65(1), pp. 95-101.

Sun, H., Lu, H., Chu, L., Shao, H. and Shi, W. (2017) 'Biochar applied with appropriate rates can reduce N leaching, keep N retention and not increase NH3 volatilization in a coastal saline soil', *Science of The Total Environment*, 575, pp. 820-825.

Sun, L., Li, L., Chen, Z., Wang, J. and Xiong, Z. (2014) 'Combined effects of nitrogen deposition and biochar application on emissions of N2O, CO2 and NH3 from agricultural and forest soils', *Soil Science and Plant Nutrition*, 60(2), pp. 254-265.

Taghizadeh-Toosi, A., Clough, T.J., Sherlock, R.R. and Condron, L.M. (2012) 'Biochar adsorbed ammonia is bioavailable', *Plant and Soil*, 350(1), pp. 57-69.

Wang, Z., Zong, H., Zheng, H., Liu, G., Chen, L. and Xing, B. (2015) 'Reduced nitrification and abundance of ammonia-oxidizing bacteria in acidic soil amended with biochar', *Chemosphere*, 138, pp. 576-583.

Yao, Y., Gao, B., Zhang, M., Inyang, M. and Zimmerman, A.R. (2012) 'Effect of biochar amendment on sorption and leaching of nitrate, ammonium, and phosphate in a sandy soil', *Chemosphere*, 89(11), pp. 1467-1471.

Yoo, G. and Kang, H. (2012) *Effects of Biochar Addition on Greenhouse Gas Emissions and Microbial Responses in a Short-Term Laboratory Experiment*.

Zhao, H., Yu, L., Yu, M., Afzal, M., Dai, Z., Brookes, P. and Xu, J. (2020) 'Nitrogen combined with biochar changed the feedback mechanism between soil nitrification and Cd availability in an acidic soil', *Journal of Hazardous Materials*, 390, p. 121631.

Zheng, H., Wang, Z., Deng, X., Herbert, S. and Xing, B. (2013) 'Impacts of adding biochar on nitrogen retention and bioavailability in agricultural soil', *Geoderma*, 206, pp. 32-39.
